# The salivary microbiota in health and disease

**DOI:** 10.1080/20002297.2020.1723975

**Published:** 2020-02-04

**Authors:** Daniel Belstrøm

**Affiliations:** Section for Periodontology and Microbiology, Department of Odontology, University of Copenhagen, Copenhagen, Denmark

**Keywords:** Saliva, microbiota, periodontitis, dental caries, systemic disease

## Abstract

The salivary microbiota (SM), comprising bacteria shed from oral surfaces, has been shown to be individualized, temporally stable and influenced by diet and lifestyle. SM reflects local bacterial alterations of the supragingival and subgingival microbiota, and periodontitis and dental-caries associated characteristics of SM have been reported. Also, data suggest an impact of systemic diseases on SM as demonstrated in patients with a wide variety of systemic diseases including diabetes, cancer, HIV and rheumatoid arthritis. The presence of systemic diseases seems to influence salivary levels of specific bacterial species, as well as α- and β-diversity of SM. The composition of SM might thereby potentially mirror oral and general health status. The contentious development of advanced molecular techniques such as metagenomics, metatranscriptomics and metabolomics has enabled the possibility to address bacterial functions rather than presence in microbial samples. However, at present only a few studies have employed such techniques on SM to reveal functional and metabolic characteristics in oral health and disease. Future studies are therefore warranted to illuminate the possible impact of metabolic functions of SM on oral and general health status. Ultimately, such an approach has the possibility to reveal novel and personalized therapeutic avenues in oral and general medicine.

## Introduction

Saliva is the fluid covering the surfaces of the oral cavity. Being instrumental in physiological processes such as mastication, swallowing and speech [1], saliva also harbors essential biological constituents including proteins and enzymes, which are essential for maintenance of oral homeostasis [2]. For example, salivary mucins and glycoproteins are the sole nutritional source in early plaque development [3], and salivary antimicrobials are critically involved in maintaining a symbiotic relationship between host and its resident microbiota [4]. This relationship is constantly stressed by internal and external ecological perturbations [5], and while the oral microbiota is resilient to minor ecological changes [6], prolonged perturbation can induce dysbiosis of the resident microbiota, which may lead to the two major oral diseases, i.e. periodontitis and dental caries [7].

By tradition microbiological analysis of patients with periodontitis has been performed on the subgingival microbiota, whereas in patients with caries, research has focused primarily on the supragingival plaque. In general medicine samples of stools are usually subject to analysis of the gastrointestinal microbiota. However, saliva is an easy and non-invasive alternative to such sampling strategies [8], and the salivary microbiota (SM) has been shown to reflect local bacterial alterations in supragingival and subgingival microbiotas [9,10]. Furthermore, oral bacterial species are reported in gut microbial samples [11,12]. Therefore, saliva might be a feasible alternative to local samples in studies of the microbiota in oral and general health and disease [13].

The purpose of the present review is to report recent research on the bacterial part of SM in oral and general health and disease, and to discuss future perspectives for this line of research. The main focuses of the present review are on the two most prevalent oral diseases i.e. periodontitis and dental caries, as well as on diabetes and cancer. The literature included is limited to studies of the bacterial part of SM using molecular techniques.

## The salivary microbiota in oral health

Saliva is sterile when being secreted into the oral cavity [14]. However, when sampled a diverse microbiota is present in saliva [15]. Accordingly, SM has been shown to be a conglomerate of bacteria shed from oral surfaces with the throat, the tongue and the tonsils as the main sites of origin [16]. The SM has been demonstrated to be individualized [17,18] and temporarily stable [19,20] in orally healthy individuals. On the other hand, circadian oscillations of SM have also been documented [21].

The composition of SM in health is to some extent shaped by environmental factors [22]. Cross-sectional data suggest that the composition differentiates in individuals living under different climate conditions [23], and a recent longitudinal study showed alterations of SM in members during an Antarctic expedition [24]. Likewise, several studies have documented an impact of diet on SM [25–27].

The SM mirrors dentate status. Accordingly, the composition of SM has been reported to differentiate between dentate and edentulous individuals [28], and full-mouth extraction impacts on SM [29]. Furthermore, SM is affected by dental developmental stages [30,31], and early life development of SM is a coordinated process, influenced by ecological perturbations such as mode of delivery, breastfeeding length and antibiotic treatment [32].

## The salivary microbiota in oral disease

### Periodontitis

The SM has been widely compared in patients with periodontitis and orally healthy controls. A substantial part of the studies has employed polymerase chain reaction (PCR)-based techniques with special emphasis on the applicability of salivary levels of specific bacterial species as a biomarker of periodontitis.

Accordingly, a number of cross-sectional PCR-based studies have compared salivary levels of putative periopathogens, especially *Porphyromonas gingivalis, Tannerella forsythia, Treponema denticola, Prevotella intermedia* and *Aggregatibacter actinomycemtomitans* in saliva from patients with periodontitis to those of orally healthy controls. A recent study from 2019 reported salivary levels of the JP2 clone of *A. actinomycemtomitans* to associate with clinical attachment loss in Moroccan adolescents [34], whereas a large-scale study comprising 977 Japanese individuals showed salivary levels of *P. gingivalis* to correlate with percentage of sites with probing pocket depth ≥4 mm [35]. In addition, a cross-sectional study of a Finnish population (n = 462) documented that combined salivary levels of *P. gingivalis, P. intermedia* and *T. forsythia* were associated with periodontitis [36]. In addition, a recent study from 2019 demonstrated higher salivary levels of two hitherto uncultured bacterial species (*Fretibacterium* sp. human oral taxon 360 and *Fretibacterium* sp. human oral taxon 356) in patients with periodontitis as compared to orally healthy controls [37]. However, no comparison of subgingival and salivary levels of the selected bacteria was performed in the above-mentioned studies, which is why the origin of periopathogens in saliva was unknown. Nevertheless, other studies have used PCR and next-generation sequencing (NGS) of the 16 rDNA gene to compare subgingival and salivary levels of putative periodontal pathogens. Taken together, these studies have demonstrated a strong correlation of subgingival and salivary levels of putative periopathogens [38–41].

Moreover, several studies have aimed to differentiate patients with periodontitis from orally healthy controls by means of salivary levels of putative periopathogens. For example, a recently published NGS-based study showed that relative abundance of *P. gingivalis* could discriminate patients with periodontitis from orally healthy controls with an AUC (area under curve) of 0.80 [42], and a PCR-based study of 9 selected periopathogens reported that it was possible to discriminate the severity of periodontitis based on salivary levels of the bacteria tested [43]. Furthermore, salivary levels of periopathogens have been used in periodontal risk assessment. For example, in a longitudinal study of 24 months duration, the combination of salivary levels of *P. gingivalis* and serum levels of *P. gingivalis’* IgG antibodies was associated with periodontal disease progression [44].

A few studies have used NGS to characterize the salivary microbiota in patients with periodontitis, and compare data with that of orally healthy controls. Accordingly, a recently published study in a Swedish cohort showed a significant periodontitis associated-microbiota with increased levels of *T. forsythia, Filifactor alocis* and *Parvimonas micra* [45]. Furthermore, several interventional studies using NGS have demonstrated an impact of non-surgical periodontal treatment on the composition of SM [9,46–48]. Interestingly, two of these studies showed a positive correlation of subgingival and salivary levels of putative periopathogens before and after periodontal treatment [9,47]. Thus, data suggest that even though periopathogens are occasionally found off the tongue [49], spill-over of bacteria from the subgingival area are probably the primary site of origin of periopathogens identified in saliva. This is why salivary levels of periopathogens might be used as a biomarker of periodontitis.

### Dental caries

The SM has been characterized in patients with severe early childhood caries (SECC), as well as in adolescent and adult populations with dental caries. In all cases, data from patients with caries have been compared to that of age-matched orally healthy controls.

Recently, two NGS-based studies performed a cross-sectional comparison of SM in patients with SECC and children <5 yrs. Without caries, and both studies reported caries-associated characteristics of SM [50,51]. Notably, co-analysis of *Candida albicans* demonstrated that carriage of *C. albicans* in children with SECC attenuated the differences observed [51]. In 2018 three longitudinal studies on SM in children with dental caries were published [52–54]. One of these studies compared their findings in patients with recurrent caries (n = 7) with those of patients with a history of caries (n = 6) and caries-free controls (n = 15). The main finding was that salivary levels of *Fusobacterium, Prevotella, Leptotrichia* and *Capnocytophaga* species could predict recurrent caries with an AUC = 0.95 [54]. Likewise, another study reported that the composition of SM in combination with information on salivary levels of host defense peptides could predict caries progression [53].

The SM in adolescents with dental caries has recently been compared to that of orally healthy adolescents [55,56]. Accordingly, an NGS-based retrospective cross-sectional study of a Swedish cohort (n = 62) showed significant caries-associated differences of SM. Specifically, higher salivary abundance of bacterial species, such as *Scardovia wiggsiae* and *Streptococcus mutans*, were identified in patients with caries. Furthermore, patients with salivary presence of *Bifidobacterium longum* had an increased caries risk [56]. In addition, a cross-sectional study of 154 adolescents confirmed distinct differences of SM in caries patients vs. healthy controls, which were partly driven by the co-occurrence of *S. wiggsiae* and *S. mutans* [55].

Increased levels of *Veillonella* and *Bifidobacterium* species were found in SM of adult patients with caries compared to that of healthy controls [57]. Likewise, a recently published study demonstrated caries-associated characteristics of SM in elderly patients with caries as compared to healthy controls [58].

Collectively, data from various NGS-based studies of the 16S rRNA gene have reported SM to differentiate patients with periodontitis and dental caries from that of orally healthy controls. Furthermore, SM differs in patients with periodontitis vs. patients with caries [59,60]. Thus, it seems that the presence of treatment requiring oral disease associates with characteristics of SM. The breakthrough of more advanced molecular techniques such as metagenomics, metatranscriptomics and metabolomics has enabled the possibility to add bacterial functions such as carbohydrate metabolism and proteolytic activity to their presence in microbial samples [61]. Accordingly, a few studies have described functional and metabolic characteristics of SM in oral health and disease [62,63]. Thus, future studies will have the possibility to focus on the possible impact of metabolic functions of the salivary microbiota as an etiological agent in periodontitis and dental caries. Such an approach may be important for the development of novel personalized therapeutic avenues.

## The salivary microbiota in systemic disease

### Diabetes and obesity

Poor oral health status associates with increased risk of systemic disease, and especially the bidirectional relationship of periodontitis and type 2 diabetes (T2DM) have been described in detail [64]. Much focus has been paid on the role of low-grade inflammation as the link between oral and systemic diseases [65]. However, the potential impact of systemic disease on SM has also been addressed.

Several studies have compared SM in patients with diabetes to that of healthy controls by means of NGS, and in general, data show that diabetes associates with a decrease in bacterial diversity of SM [66–68]. In addition, higher salivary levels of *P. gingivalis, T. forsythia* and *F. alocis* were reported in patients with gestational diabetes [69], whereas only minor differences were identified in children with T2DM, when compared to obese and healthy controls, respectively [70].

Also, SM in obese individuals has been compared to that of lean controls. A metagenomic study from 2018 showed decreased bacterial diversity and richness in saliva from obese individuals. Furthermore, functional analysis documented higher bacterial expression of immune disease-related genes in obese individuals [71]. Likewise, obesity was reported to modify salivary bacterial diversity in patients with T2DM [72]. In addition, a PCR-based analysis demonstrated higher salivary levels of *P. gingivalis, T. forsythia* and *Fusobacterium nuclatum* in obese patients with and without T2DM, as compared to lean controls [73].

Finally, a potential impact of salivary glucose concentration on SM has been investigated. Specifically, DNA-DNA hybridization of >2500 saliva samples collected from Kuwaiti children showed high salivary glucose concentration to associate with a decrease in bacterial diversity [74], and NGS of SM revealed increased abundance of *Leptotrichia, Staphylococcus, Catonella* and *Bulledia* species in individuals with impaired fasting glucose [75].

Thus, data suggest that T2DM, obesity and poorly controlled clearance of glucose i.e. impaired fasting glucose is associated with comparable impacts on SM.

### Cancer

The SM has been studied in patients with oral squamous cell carcinoma (OSCC). One large-scale report characterized SM in 138 patients with OSCC by means of NGS and compared data with that of saliva from 151 matched healthy controls. The main finding was higher salivary abundance of periodontitis-associated species, such as *Prevotella tannerae, F. nucleatum*, and *P. intermedia* in patients with OSCC [76]. Likewise, two studies have reported SM to differentiate in patients with oral leukoplakia [77] and patients with other epithelial precursor lesions to OSCC [78], when compared to that of orally healthy controls. It is, however, more interesting that specific alterations of SM were present in samples from OSCC patients versus patients with leukoplakia and epithelial precursor lesions. Specifically, higher salivary levels of *Solobacterium* species and lower levels of *Streptotoccus* species were recorded in OSCC patients compared to leukoplakia patients [77], whereas salivary abundance of *Bacillus, Enterococcus, Parvimonas, Peptostreptococcus* and *Slackia* species discriminated patients with OSCC from patients with epithelial precursor lesions [78]. Thus, cross-sectional data suggest that SM might be used in screening of OSCC. However, one study has shown that the microbiota identified in OSCC lesions is different from the concomitant SM in patients with OSCC [79]. Thus, prospective data are warranted to evaluate if the specific strains of SM are causally involved in the development of OSCC.

The effect of OSCC treatment i.e. radiotherapy and chemotherapy on SM has been longitudinally evaluated. One study showed that radiotherapy was associated with an increase in salivary levels of *Lactobacillus* species, which was reversed to baseline levels 1 year after radiotherapy. Interestingly, strong correlation was observed in salivary levels of *Lactobacillus* species and fluctuations of saliva flow rates and salivary pH levels during radiotherapy [80]. Another report demonstrated that chemotherapy-induced oral mucositis was associated with a decrease in salivary levels of health-associated bacterial genera, including *Streptococcus, Actinomyces* and *Veillonella* in combination with an increase of the periodontitis-associated genera *Fusobacterium* and *Prevotella*, and increased transcription of genes related to innate immunity and apoptosis in oral epithelial cells from patients with oral mucositis [81].

Cross-sectional studies have evaluated SM in patients with cancers outside the oral cavity. Accordingly, high salivary levels of *T. forsythia* and *A. actinomycetemcomitans* and low bacterial diversity were reported in patients with precancerous gastric lesions [82]. On the other hand, gastrointestinal cancers of various origins were associated with an increased salivary bacterial diversity and high levels of *P. gingivalis* as compared to matched healthy controls [83]. In line, a study from 2019 used NGS of SM to discriminate patients with throat cancer from healthy controls and patients with vocal cord polyps with an AUC = 0.87 [84]. Finally, two cross-sectional studies have compared SM in patients with lung cancer to healthy controls. The main findings were higher levels of *Streptococcus* and *Veillonella* species in saliva from patients with non-small cell lung cancer [85], and lower salivary levels of *Streptococcus* species in combination with low bacterial diversity in female non-smokers with lung cancer as compared to matched healthy controls [86].

Therefore, data suggest that various non-oral cancers are associated with different alterations of SM. Data is, however, based solely on cross-sectional studies, which hampers the possibility to draw any conclusions on causality at present.

### Other systemic diseases

Periodontitis has several important comorbidities such as rheumatoid arthritis and atherosclerosis [65]. Interestingly, SM has been reported to differ in patients with such comorbidities as compared to healthy controls. For example, rheumatoid arthritis associated with dysbiosis of SM with depletion of *Haemophilus* species in saliva, dental plaque and fecal samples, which was partly normalized by treatment of rheumatoid arthritis [87]. In addition, salivary levels of four periopathogens, i.e. *P. gingivalis, T. denticola, T. forsythia* and *P. intermedia*, have been suggested to be independently involved in lowering serum levels of high-density lipoproteins, which may be associated with an increased risk of atherosclerosis [88].

Immune defects have an impact on SM. Accordingly, immune deficiency as expressed by the manifest human immunodeficiency virus (HIV) has been reported to influence α- and β-diversity of SM [89]. Furthermore, increased salivary levels of periopathogenic species including *Prevotella melanogenica* and *Rothia mucilaginosa* were shown to correlate with the extent of CD4 + T cell depletion in patients with HIV [90].

In several of the above-mentioned studies, correlation changes to the salivary and fecal microbiotas were evident [87,89], which highlight the possibility to use saliva-based screening as a substitute to fecal samples in microbiologic studies of systemic diseases. An example of such an approach was published in a recent longitudinal study, which linked dysbiosis of SM during the first 7 years of life with development of allergy [32].

## Future perspectives

The accumulated evidence suggests that SM is individualized and relatively stable over time as long as oral and general health is maintained. In addition, local bacterial changes associated with periodontitis and dental caries are reflected by alterations of SM. Furthermore, presence of systemic disease appears to have an impact on SM. Thus, SM seems to reflect oral and general health status. However, future studies are needed to reveal if changes of SM precede clinical signs of disease, which would enable the possibility to use SM in the prediction of future disease risk. Ideally, this could be performed targeting subtypes or strains of specific bacterial species in SM such as *P. gingivalis* or *A. actinomycemcomitans* [91] in periodontits or *S. mutans* in caires. An elegant example of such an approach was recently published in a study showing that analysis of *S. mutans* in saliva based on adhesion subtypes could be used for future caries risk prediction [92]. It is also interesting that in a recent cross-sectional study from 2017 data showed that orally healthy individuals can be divided into five ecotypes based on characteristics of SM, the salivary metabolome and host-related biochemical salivary parameters [93]. In addition, a recent longitudinal study in hospitalized cancer patients demonstrated that increased variability of SM was associated with adverse clinical outcomes [94]. Notably, these studies demonstrate the possibility to use SM in risk assessment and treatment evaluation of oral and systemic disease. Accordingly, prospective longitudinal studies are urgently needed to reveal the full potential of using SM in the field of precision medicine.
